# Patient experiences of interprofessional collaboration and intersectoral communication in rare disease healthcare in Germany – a mixed-methods study

**DOI:** 10.1186/s13023-024-03207-9

**Published:** 2024-05-13

**Authors:** Laura Inhestern, Ramona Otto, Maja Brandt, David Zybarth, Ralf Oheim, Helke Schüler, Thomas S. Mir, Konstantinos Tsiakas, Payam Dibaj, Jana Zschüntzsch, Pamela M. Okun, Ute Hegenbart, Olaf Sommerburg, Christoph Schramm, Christina Weiler-Normann, Martin Härter, Corinna Bergelt

**Affiliations:** 1 Department of Medical Psychology, University Medical Center Hamburg-Eppendorf, Hamburg, Germany; 2 Institute of Osteology and Biomechanics, University Medical Center Hamburg-Eppendorf, Hamburg, Germany; 3 Department of Cardiology, University Medical Center Hamburg-Eppendorf, Hamburg, Germany; 4 Department of Pediatric Cardiology, University Medical Center Hamburg-Eppendorf, Hamburg, Germany; 5 University Children’s Hospital, University Medical Center Hamburg-Eppendorf, Hamburg, Germany; 6 Center for Rare Diseases Göttingen (ZSEG), Department of Pediatrics, University Medical Center Göttingen, Göttingen, Germany; 7 Department of Neurology, University Medical Center Göttingen, Göttingen, Germany; 8 Center for Rare Diseases Heidelberg, Medical Center, University of Heidelberg, Heidelberg, Germany; 9 Amyloidosis Center, Heidelberg University Hospital, Heidelberg, Germany; 10 Division of Pediatric Pulmonology, Allergy, and Cystic Fibrosis Center, Department of Pediatrics III, Heidelberg University Hospital, Heidelberg, Germany; 11 Translational Lung Research Center Heidelberg (TLRC), German Center for Lung Research (DZL), Heidelberg, Germany; 12 Martin Zeitz Center for Rare Diseases, University Medical Center Hamburg-Eppendorf, Hamburg, Germany; 13 Department of Medical Psychology, University Medicine Greifswald, Greifswald, Germany

**Keywords:** Rare disease, Healthcare, Intersectoral collaboration, Patient satisfaction, Quality of care

## Abstract

**Background:**

Rare diseases are often complex, chronic and many of them life-shortening. In Germany, healthcare for rare diseases is organized in expert centers for rare diseases. Most patients additionally have regional general practicioners and specialists for basic medical care. Thus, collaboration and information exchange between sectors is highly relevant. Our study focuses on the patient and caregiver perspective on intersectoral and interdisciplinary care between local healthcare professionals (HCPs) and centers for rare diseases in Germany. The aims were (1) to investigate patients’ and caregivers’ general experience of healthcare, (2) to analyse patients’ and caregivers’ perception of collaboration and cooperation between local healthcare professionals and expert centers for rare diseases and (3) to investigate patients’ and caregivers’ satisfaction with healthcare in the expert centers for rare diseases.

**Results:**

In total 299 individuals of whom 176 were patients and 123 were caregivers to pediatric patients participated in a survey using a questionnaire comprising several instruments and constructs. Fifty participants were additionally interviewed using a semistructured guideline. Most patients reported to receive written information about their care, have a contact person for medical issues and experienced interdisciplinary exchange within the centers for rare diseases. Patients and caregivers in our sample were mainly satisfied with the healthcare in the centers for rare diseases. The qualitative interviews showed a rather mixed picture including experiences of uncoordinated care, low engagement and communication difficulties between professionals of different sectors. Patients reported several factors that influenced the organization and quality of healthcare e.g. engagement and health literacy in patients or engagement of HCPs.

**Conclusions:**

Our findings indicate the high relevance of transferring affected patients to specialized care as fast as possible to provide best medical treatment and increase patient satisfaction. Intersectoral collaboration should exceed written information exchange and should unburden patients of being and feeling responsible for communication between sectors and specialists. Results indicate a lack of inclusion of psychosocial aspects in routine care, which suggests opportunities for necessary improvements.

## Introduction

According to the European Union, diseases affecting not more than 5 per 10 000 people are classified as rare diseases [1]. Even though every single rare disease only affects a relatively small number of patients, taken together, rare diseases affect up to 5.9% of the population [2]. Correspondingly, about 18–30 million persons in the EU and 262–446 million persons globally [2] are affected by one of the 6.000–8.000 known rare diseases [3]. However, estimations about prevalence have been discussed to be over- or underestimated [4].

Still, most rare diseases are complex and chronic, and many of them life-shortening [5]. Although rare diseases can be very heterogeneous in their clinical manifestation, patients face many common challenges due to the rarity of their condition. Besides delayed diagnoses by up to eight years in average [6], frequent healthcare problems for patients with rare diseases are lack of treatment options [7], insufficient knowledge and expertise in health care professionals (HCPs) and difficult access to specialized care. Moreover, many rare diseases affect more than one organ system and, hence, adequate treatment requires several specialists [8]. In Germany, specialized medical care for patients with rare diseases is organized in a model of care delivery based in centers for rare diseases [9, 10]. These centers comprise three levels based on certain criteria [10]: Reference centers (so called A-centers) provide interdisciplinary structures for patients with undiagnosed or unclear rare diseases, conduct basic and clinical research, provide education for students and HCPs and comprise at least five centers specialized for certain disease groups. These specialized centers (B-centers) provide inpatient and outpatient care for certain disease groups, are integrated into a hospital setting and conduct basic and clinical research. Cooperating centers (so called C-centres) are specialized for certain disease groups and provide outpatient care. In 2021 the certification process of reference centers for rare diseases in Germany has been implemented [11]. There are currently 37 A-centers listed in the *se-atlas*, a German web-based information platform for rare diseases [12]. However, most these have not been certified yet. Most of these centers are part of university medical centers and hence tend to be located in metropolitan areas of Germany. This structure can be problematic for patients living in more rural areas, as they can have long journeys to access specialized care while the implementation of telemedicine is still rudimentary in Germany [13, 14]. Regional accessibility of specialized rare disease healthcare is thus another problem for several patients and their relatives.

All of the presented aspects support the necessity of interdisciplinary cooperation and intersectoral communication between HCPs. The separation of healthcare sectors poses an additional problem, e.g., difficulties in information exchange between different healthcare providers or inadequate dissemination of the electronic patient record [15, 16]. Since rare disease healthcare commonly needs to involve specialized physicians from different fields and healthcare sectors, intersectoral collaboration concepts are required to enable and maintain information exchange.

In terms of a patient-centred approach, patients’ perspective on intersectoral collaboration and communication is of high importance. Patients and caregivers have raised the demand for interdisciplinary health care teams that are well coordinated, patient- and family-centred and support navigation through health care [17–19].

Our study focuses on the patient and caregiver perspective on intersectoral and interdisciplinary healthcare between local HCPs and centers for rare diseases in Germany. The aims were (1) to investigate patients’ and caregivers’ general experience of healthcare, (2) to analyse patients’ and caregivers’ perception of collaboration and cooperation between local healthcare professionals and expert centers for rare diseases and (3) to investigate patients’ and caregivers’ satisfaction with healthcare in the expert centers for rare diseases.

## Methods

The presented study was part of a multiperspective mixed methods study to investigate concepts for intersectoral collaboration in healthcare of people living with rare diseases [20]. Based on an assessment of concepts for intersectoral collaboration and communication of expert centers for rare diseases, we conducted a mixed-methods survey with patients and caregivers from German centers for rare disease with most convincing concepts.

### Design and participants

We conducted a cross-sectional mixed-methods survey with patients and caregivers of pediatric patients with rare diseases. Participants were recruited from January 2021 to January 2022 via two different approaches. First, six centres for rare diseases were selected for recruitment based on a positive assessment of their concept for intersectoral collaboration and communication. Three of the six selected centres actually participated in the recruitment of patients and caregivers of pediatric patients. Secondly, patients and caregivers were recruited by cooperating patient organizations, who informed their members about the study. The following inclusion criteria were defined: diagnosed rare disease or reasonable suspicion of a rare disease diagnosis, consent to participate, treatment in a center for rare diseases. Exclusion criteria were insufficient knowledge of German to complete the questionnaire, cognitive impairment (as assessed by the HCP), too much burden to participate (as assessed by the HCP) or no interest.

Centres for rare diseases received prepared study material for the participants (incl. study information, informed consent form, questionnaire, franked return envelope to the study team). In the centers, the coordination centre (A-centre) and specialized clinics selected by the centers (B-centers) invited their patients resp. caregivers of their pediatric patients to participate in the study and disseminated the study material. Patients and caregivers filled in the questionnaire and sent it directly to the study team using the return envelope. Patients and caregivers who were informed about the study by the patient organizations, contacted the study team proactively. If interested, they received all study material by mail.

In the study information, survey participants were also invited to participate in a semistructured interview and to provide their contact data in case of interest. Survey participants were then contacted by the study team and an interview appointment was set. Interviews were conducted by telephone by one of the study team members (DZ (psychologist), MB (psychologist), ROt (health science); all M.Sc., sufficiently trained in semi-structured interviews and supervised by LI). Our aim was to include *n* = 50 patients/caregivers to reach theoretical saturation in the qualitative interviews.

### Instruments

The survey was conducted as a paper-pencil-questionnaire covering a set of questions, e.g. the experiences of intersectoral communication, as well as existing and validated instruments on patient satisfaction, satisfaction with healthcare, psychosocial burden, quality of life and needs/unmet needs. In addition, relevant data regarding disease and healthcare history were assessed based on self-report. Following the research objectives, the study focused on the following variables and instruments.

#### Demographic and disease-related data

We assessed age, sex (male, female, divers), nationality and socio-ecomonic status according to the Winkler and Stolzenberg-Index [21]. Additionally, disease diagnosis and time since diagnosis were assessed.

#### Experiences of healthcare and intersectoral collaboration and communication

To assess experiences of healthcare and intersectoral collaboration and communication, we developed a set of questions based on literature review and the assessment of concepts for intersectoral collaboration and communication of expert centers for rare diseases previous to the survey. Patients and caregivers were asked about their access to specialised healthcare services for their rare disease (e.g. How many medical facilities have you visited regarding the diagnosis of rare disease/symptoms? Which medical facilities have you visited? How do you rate the time of transfer to the center for rare diseases?). Regarding their experiences in the centers for rare diseases, patients and caregivers answered questions on their contact to the center, their information resources, their referral to the center (incl. necessary medical records), their access to the center (distance, waiting time) and communication with the center.

#### Satisfation with healthcare

Satisfaction with healthcare in the centers for rare diseases and specialized medical care was assessed using the German questionnaires ZAPA and ZUF-8 [22, 23]. We adapted the instructions of both questionnaires and asked patients and caregivers to focus on the care in the centers for rare diseases or specialized centers where they are mainly treated for their rare disease. ZAPA (Fragebogen zur Zufriedenheit in der ambulanten Versorgung) is a short instrument to assess satisfaction with outpatient care and consists of four items which can be rated on a 4-point-Likert scale. The sum score can be transformed in a scale ranging from 0 (lowest level of satisfaction) to 100 (highest level of satisfaction). The ZUF-8 (Fragebogen zur Messung der Patientenzufriedenheit) assesses patient satisfaction with inpatient care and consists of eight items with a 4-point-Likert scale. A sum score ranging from 8 (lowest level of satisfaction) to 32 (highest level of satisfaction) can be calculated.

#### Qualitative interview guideline

For the qualitative interviews, a semi-structured guideline was developed to target all relevant areas concerning disease and healthcare history, experiences of intersectoral collaboration and communication, patient satisfaction as well as suggestions for improvement (Table 1).

**Table 1:** Topics of the interview guideline

Topic	Examplary questions
Experiences before visiting the center for rare diseases	• When did the first symptoms occur? • How did you experience the time from first symptoms to the transfer to the center for rare diseases?
Access to the center for rare diseases	• When and how did you get to know about centers for rare diseases? • How did the transition to the center proceed?
Experiences of healthcare in the center for rare diseases	• How did/do you experience diagnostic processes and treatment within the center? • How is your general practitioner (GP)/pediatrician involved in the treatment?
Experiences of collaboration	• How did/do you experience collaboration between ○ GP/pediatrician and center for rare disease? ○ GP and other specialists?
Possibilities for improvement	• What kind of support would have been necessary retrospectively? • What would be the best way for designing healthcare from first symptoms to treatment?
Others	• What additional support offers did you/do you use? • How was/is your contact to patient organizations/self-help groups?

### Analyses

Quantitative data was analysed using descriptive statistics. Mean and standard deviation was used for metric data, frequency and percentages were used for categorical data. In case of incomplete responses, we analyzed the completed responses, leading to variant sample sizes for single variables. We conducted t-tests for group comparisons of patients and caregivers. Variance homogeneity between groups was tested using Levene-Test. In case of heterogeneity, we applied Welch-correction. Quantitative data was analysed using SPSS Statistics Version 27.

For the qualitative analyses, interviews were transcribed verbatim. Transcripts were not returned to interviewees for comments or corrections. Qualitative data was analysed using qualitative content analysis [24]. Categories were generated based on the interview guideline and previous considerations (theoretical and derived from the first study phase) and based on the transcripts. A coding guideline including categories, coding rules and anchor examples was elaborated. After that, the interviews were coded by two team members (ROt coded *n* = 24 interviews, CO coded *n* = 26 interviews). Interview duration was M = 43 min (SD = 14.0). Qualitative analysis was conducted with MAXQDA software.

Qualitative and quantitative findings were synthesized according to the research questions by the study team.

## Results

### Participants

In total 299 individuals participated in the quantitative study of whom 176 were patients themselves and 123 were caregivers to pediatric patients. The majority of participants (79%) were recruited by the pre-selected best-practice centers for rare diseases, the remaining 21% were reached by patient organizations.

Overall, 66% of the participants were female (84% mothers in the subgroup of caregivers). The average age was 45.2 years (SD = 12.3 years) in the total sample, with caregivers being on average younger than patients (M = 39.8 years, SD = 7.1 years vs. M = 48.9 years, SD = 16.7 years). 98% were German and the vast majority of the cohort (90%) could be assigned to middle or upper class according to Winkler et al. [21]

The most frequent diagnoses in the study population were the Marfan Syndrome (20%) and Esophageal Atresia (15%). Almost 23% of the study population was still in the diagnostic process at the time of the survey. Mean time since diagnosis was 9.9 years (SD = 11.2).

In total, 50 survey participants additionally participated in an interview. In this subsample 38 were patients and 12 particpants were caregivers of pediatric patients. 66% were female and mean age was M = 50. The most frequent disease groups among the interviewees were rare metabolic diseases (29%), rare diseases of the connective tissue (25%), and rare musculoskeletal diseases (19%). 10% of the participants were still in the diagnostic process at the time of the interview.

### Experiences of healthcare

#### Access to specialized care in centers for rare diseases

Most participants (68%) reported that their attending physician supported their referral to a center for rare diseases by providing necessary documents and information. In 28%, the referring physician made the appointment in the center for rare diseases. Of those participants being referred to a center for rare diseases, 66% perceived the timing as appropriate, whereas 24% assessed the timing of referral as too late or far too late.

One out of five participants perceived their physicians’ general familiarity with centers for rare diseases as insufficient. Most relevant information source for patients and caregivers about the centers of rare diseases was the homepage of the centers. Participants reported to have contacted the center for rare diseases by telephone or email. Although waiting time for a first appointment was up to several months, most of the participants perceived the waiting time as reasonable (93%).

In the qualitative interviews, participants reported a spectrum of experiences about the access to specialized care. One central impeding factor was the unknown diagnosis, which was still not clarified for 5 of 50 interviewees at the time of the interview. Some interviewees reported long periods of waiting time for appointments, examinations and examination results in general. Besides long waiting time, diagnostic examinations were not coordinated between different practitioners and professions, leading to double examinations. These aspects had not only prolonged the diagnostic process, but also lead to additional psychologic strain.

*„The waiting time for the first appointment was very long. You had to try to forget about it until the month came and you saw it in the calendar. Nine months is a very, very long time.”*

(20-B02-I06-B)

Further factors influencing the diagnostic process and the access to specialized care reported in the interviews were wrong diagnoses, lack of knowledge or HCPs exceeding their competencies. Positive factors were timely appointments and structured processes, knowledge about the landscape of healthcare (including centres for rare diseases), interdisciplinary exchange as well as engagement of single practitioners and patients themselves.

#### Availability and organization of the centers for rare diseases

33% of the participants of the quantitative survey reported to travel more than 100 km to their specialized care center, 36% experience the way as rather or very stressful. The majority of participants in the quantitative survey reported to have a direct contact person for medical concerns, organizational issues or both. However, 23% reported not having the contact details of this person. About 20% reported not having any contact person in charge of them.

75% of the participants felt that the center for rare diseases was available if they had urgent concerns. Most participants were involved in diagnostics and treatment planning (95%) and perceived the information about their diagnosis as sufficient and appropriate. In their specialized care centre, almost all patients and caregivers had personal contact in terms of diagnostic appointments, information appointments, treatment planning or check-up appointments. According to the participants, the centers were competent in handling their rare diseases. Over 63% agreed that the professionals in the centers helped them to manage their situation better. Three-quarters agreed that the centers were handling their situation differently than other medical institutions and 93% had the impression that the professionals in the centers understood their situation well. 86% trusted that the ongoing (medical) measures would help their situation.

Most participants received a note, epicrisis or written recommendations for further treatment (87%) after consultation; of those, 83% reported having discussed the content with their treating physicians in the centre. However, 22% reported to still having open questions after appointments.

In the interviews, participants described different experiences concerning the availability and organization of the centers. Some patients and caregivers of pediatric patients mentioned limited options to contact the centers. One central aspect emerging from the interviews was the relevance of continuity within the healthcare team. According to the interviewees, it is beneficial when physicians handle the patients’ treatment over a long period, whereas frequent changes of healthcare team members would lead to difficulties such as loss of information or lack of responsibility.

*„That one physician still treating me, I am very sure she knows the direction we should head to. She is also my continuos contact person and that is all right. I am scared of the day she might leave. I just feel safe with her.” (23-B01-I08-B)*.

Further positive aspects on the healthcare within the centers were communication and integration of patients in the healthcare process and well-established information exchange among the specialists in the center.

#### Intersectoral collaboration and cooperation

59% of the participants received care of their rare disease by the center for rare disease and at least one resident doctor; 27% reported that only the centre for rare disease was in charge regarding the rare disease. 38% of the participants reported no direct exchange between the center for rare diseases and their resident physician (33% reported there was exchange, 30% do not know). In case of exchange, 50% believed that the resident physician was involved in the care planning and healthcare of the rare disease.

25% of the participants reported having received offers of psychosocial support through the center for rare diseases, 50% used this offer. Of those who did not get the offer, 33% wished to get support. 43% received information about patient organizations or self-help groups from the center for rare diseases. Of those who did not recieved the information, 42% would have wished for such information. Every second participant was in touch with a patient organization at least once (13% based on recommendations from the center for rare diseases, 36% on their own initiative).

Whereas most interviewees reported well-functioning communication structures within the centers for rare diseases, they described a lack of interdisciplinary and interprofessional communication as well as between healthcare sectors. According to interviewees, centers for rare diseases and resident physicians or specialists generally communicate in written form (e.g. epicrisis, medical reports) leading to time delays of several months. In many cases, the patients or caregivers perceived themselves as communicators between sectors transferring relevant information and/or delivering documents in time.

*“I have to coordinate this all by myself. I have all my medical reports and diagnostic findings, I have a whole file of them. I have all of this here and I coordinate who gets the reports and I forward them proactively. This is how it always works, either I take charge of it or nothing happens.” (20-A01-I01-B)*.

### Satisfaction with healthcare in centers for rare diseases

The majority of participants reported high levels of satisfaction with the care they received in the centers for rare diseases (Table 2). Regarding the overall satisfaction with healthcare of the rare disease, 83% reported to be rather or highly satisfied, whereas 17% were rather or absolutely dissatisfied. Most participants were rather or highly satisfied with the communication between members of the healthcare team (78%). There were no significant differences in overall satisfaction between patients and caregivers. In the satisfaction questionnaire, statistically significant differences were identified on item level (Table 2).

**Table 2:** Patient and caregiver satisfaction with healthcare of the rare disease (*n* = 299)

Patient satisfaction	Total sample	Patients	Caregivers	t(df), *p* (d)^3^
	M, SD	M, SD	M, SD	
ZAPA ^1^ (0 = not at all satisfied, 3 = absolutely satisfied)				
Do you trust the doctors in the center?	2.6, 0.6	2.6, 0.6	2.7, 0.5	t(284)=-0.835, *p* = .405
How satisfied are you in general with the doctors in terms of the quality and amount of information they received?	2.6, 0.6	2.5, 0.7	2.7, 0.5	t(276)=-2.606, *p* = .010 (d = 0.30)
How satisfied are you in general with the doctors in relation to your participation in medical decisions?	2.6, 0.6	2.6, 0.7	2.7, 0.6	t(273)=-1.487, *p* = .138
How do you rate the quality of the treatment given by the doctors in general?	2.7, 0.6	2.6, 0.6	2.7, 0.5	t(273)=-2.175, *p* = .030 (d = 0.25)
Transformed total scale (0-100)^2^	87.2, 17.3	85.5, 18.4	89.6, 15.2	t(273)=-1.996, *p* = .040 (d = 0.24)
ZUF-8^1^ (1 = not at all satisfied, 4 = absolutely satisfied)				
How would you rate the quality of the healthcare you have received?	3.5, 0.6	3.4, 0.6	3.7, 0.5	t(275)=-3.655, *p* < .001 (d = 0.43)
Did you get the kind of healthcare you wanted?	3.5, 0.6	3.4, 0.6	3.6, 0.5	t(270)=-3.926, *p* < .001 (d = 0.46)
To what extent did the healthcare meet your needs?	3.4, 0.7	3.4, 0.7	3.5, 0.6	t(280)=-2.041, *p* = .042 (d = 0.25)
If a friend were in need of similar help, would you recommend the healthcare to him or her?	3.8, 0.5	3.7, 0.6	3.8, 0.4	t(281)=-1.659, *p* = .098
How satisfied are you with the amount of help you have received?	3.4, 0.7	3.4, 0.6	3.5, 0.8	t(281)=-0.600, *p* = .561
Has the healthcare you received help you deal more effectively with your problems?	3.5, 0.7	3.5, 0.7	3.6, 0.6	t(269)=-2.272, *p* = .024 (d = 0.27)
In an overall general sense, how satisfied are you with the healthcare you have received?	3.6, 0.6	3.5, 0.6	3.7, 0.6	t(264)=-2.565, *p* = .011 (d = 0.30)
If you were to seek help again, would you come back to the center?	3.8, 0.5	3.8, 0.5	3.9, 0.4	t(282)=-1.006, *p* = .3015
Sum score (8 = lowest satisfaction, 32 = highest satisfaction)	28.6, 3.7	28.0, 3.9	29.3, 3.2	t(274)=-3.076, *p* = .002 (d = 0.36)
How satisfied are you with the overall healthcare of the rare disease you/your child receives?	3.2, 0.8	3.2, 0.9	3.3, 0.7	t(286)=-1.700, *p* = .090
How satisfied are you with the communication between healthcare professionals, who are involved in your/your child’s healthcare?	3.1, 0.9	3.1, 0.9	3.2, 0.9	t(284)=-1.083, *p* = .280

ZAPA, *Fragebogen zur Zufriedenheit in der ambulanten Versorgung*, a short instrument to assess satisfaction; ZUF-8, *Fragebogen zur Messung der Patientenzufriedenheit*, a short instrument to assess patient satisfaction with care

^1^ participants received instruction to focus on the care in the centers for rare diseases or specialized centers where they are treated for their rare disease, ^2^ Transformed sum score of the ZAPA: 0 = lowest satisfaction, 100 = highest satisfaction, ^3^ t-test for independent samples, in case of heterogeneity of variances (Levene-Test) Welch-correction was used, in case of statistical significance (two-sided, alpha < 0.05), Cohen’s d is reported (small effect: d = 0.2, medium effect d = 0.5, large effect d = 0.8)

### Additional aspects of healthcare

In the interviews, patients and caregivers reported additional aspects to be relevant regarding their healthcare. They described a lack of knowledge of HCPs about rare diseases and about specifics of their disease to consider in therapies such as speech therapy, physiotherapy or occupational therapy. Moreover, difficulties with insurances (health, pension or care insurance) and financial difficulties due to lack of coverage of necessary costs for their healthcare are reported.

## Discussion

This study investigated the experiences of patients and caregivers of pediatric patients in routine healthcare in centers for rare diseases. The study provides insights into the challenges of patients and caregivers navigating through the German healthcare system. Our study sample included patients, suffering from various rare diseases from different regions in Germany and thus provides an impression of patients’ shared experiences in healthcare for rare disease. Following a mixed-methods approach, findings from our quantitative survey are complemented by detailed findings from the interview data and hence enhance the understanding of the situation of affected patients.

Patients and caregivers of pediatric patients in our sample were mainly satisfied with their healthcare. As recruitment strategy included a pre-selection of centers for rare diseases with functioning concepts for intersectoral collaboration and communication based on self-report of the centers, our findings confirm this self-evaluation from the patient perspective. Most patients reported to receive written information about their care, have a contact person for medical issues and experienced interdisciplinary exchange within the centers for rare diseases. These aspects of care can reduce the negative impact experienced by patients [25]. Transferring functional concepts, such as establishing a communication structure with patients, providing patient-centred information, and interdisciplinary exchange, could improve care across different specialist outpatient clinics or centres. Interdisciplinary exchange should be organized at local and national level. Particularly in the case of rare diseases international exchange is important for advancing research and healthcare. The European Reference Networks for Rare Diseases can provide a suitable platform for this (https://health.ec.europa.eu/european-reference-networks_en).

Our findings demonstrate that established structures in specialized care can lead to high patient satisfaction. However, the qualitative interviews revealed a rather mixed picture including experiences of uncoordinated care, low engagement and communication difficulties between professionals. The results indicate that high engagement and health literacy in patients is required to timely transfer relevant information between health care sectors. Moreover, engagement and experiences of single professionals were supportive. These findings underline that attitudes, abilities and opportunities are crucial barriers and facilitators for care coordination, which has been postulated in a recent study [26].

Established structures within the centers of rare diseases are recognized by the patients and evaluated positively. As supported by our findings, many patients have had experienced long periods of unclear diagnosis. A lack of knowledge in physicians about rare diseases and centers for rare diseases was described by our study participants [27]. It seems that being referred to a specialized center for rare diseases leads to a high quality of healthcare and increased patient satisfaction [28]. This supports the necessity of raising the awareness for potential rare diagnoses in resident practitioners and the awareness for proactive referral in cases of suspected rare diseases. Similar results were found by Simpson and colleagues, who reported that a lack of coordination between resident physicians, specialists and other professionals lead to delays in diagnosis and in access to care [25].

Regarding intersectoral collaboration, most communication between healthcare professionals was reported to be in writing (e.g. medical reports) and with a time delay. Patients and caregivers reported often to be the central person proceeding information and coordinating care between specialized centers and local healthcare professionals. Whereas the interdisciplinary exchange within the centers seemed to work appropriately, the communication with professionals outside of the center was rated as insufficient. Since general practitioners play an important role in the daily care of patients with rare diseases [29], more collaboration and communication is highly indicated. This supports the need for establishing structures to unburden patients and caregivers from long distance travelling and being responsible for organizing their own healthcare [27]. This unmet need has already been recognized for some rare disease groups and resulted in pilot projects aiming to transfer patient guides to standard care in Germany [30]. The patient guides work as case managers and significantly improve the situation for people with the disease. They act as contact persons, mediators and coordinators within the respective facility, organize interdisciplinary consultations, guide the patient to the appropriate services and take over interface communication with the outpatient sector [30]. Similarly, the project Innovcare (https://innovcare.eu) developed a holistic care pathway to enable coordination between “health, social and local services to improve care”, which has been piloted in Romania and showed particular impact on patient’s empowerment, information and self-confidence (https://innovcare.eu/wp-content/uploads/2018/12/INNOVCare_WP7_Evaluation-report_final-version.pdf).

Psychological and social support have been identified as central unmet needs in patients with rare diseases and their families [27, 31]. Our findings indicate that these aspects are not routinely addressed in German centers for rare diseases. Besides medical professionals, further disciplines such as physiotherapy, occupational therapy or dieticians are included in healthcare of patients with rare diseases [32]. As coordination of these specialists needs further resources of patients and caregivers, further investigation and interventions on care coordination should also include these aspects.

### Limitations

One limitation of our study is the recruitment strategy. Due to the study design and objectives, most participants were recruited by three of six pre-selected centers for rare diseases based on their concept to manage intersectoral collaboration and communication [20]. Hence, most participating patients and caregivers received specialized medical care. Moreover, as in Germany a center structure in healthcare of people with rare diseases has been established, our findings might not be applicable for patients from other healthcare systems in other countries. Future research should include a selected sample of centers that is not pre-selected to compare centers with established intersectoral collaborations and communication to those without established practices. Moreover, comparing healthcare for rare diseases internationally (including those without established centers for rare diseases) can facilitate the derivation of best practices across healthcare systems.

As a majority of caregiving participants are mothers, it seems to be difficult to draw conclusions on the situation of fathers as well. The underrepresentation of this special group is a common problem in similar studies (https://www.eurordis.org/publications/juggling-care-and-daily-life-the-balancing-act-of-the-rare-disease-community/). We assessed solely the subjective perspective of the patients and caregivers on experiences and satisfaction. These constructs may not always be clearly distinctive and to address the specific study aims, items on patient experiences have been self-develop without previous validation studies. We did not systematically investigate the reliability of the codings in our qualitative study.

With regard to sample characteristics, predominantly patients and caregivers with middle to high socio-economic status and German nationality participated in our study. Hence, we cannot draw any conclusions on patients from low socio-economic status, who might experience healthcare differently. Moreover, we cannot exclude any effects of social desirability since patients and caregivers were mainly invited for participation by their specialists in the centers for rare diseases. The sample composition between the quantitative and qualitative studies differs, e.g. with regard to diagnosis, age or ratio of caregivers and patients. Hence, there may be a bias in the perception of healthcare. However, we cannot analyse potential differences systematically.

The study was conducted during the beginning of the Covid-19 pandemic. Throughout this period lots of organizational changes were made in medical facilities in order to minimize infection risks for patients and caregivers. This additional burden might have led to a decreased willingness for study participation. Furthermore, the study aimed at investigating organizational processes related to communication and intersectoral collaboration. As these processes were more likely to be changed in this phase of the pandemic, our results might differ from results gained in pre- and post-pandemic routine.

## Conclusion

Our findings suggest that patients and caregivers experience healthcare in selected centers for rare diseases mainly as positive. This indicates the high relevance of transferring affected patients to specialized care as fast as possible to provide best medical treatment and facilitate patient satisfaction. Intersectoral collaboration and communication should exceed written information exchange and should unburden patients of being forced to act as a communicator between sectors and specialists. Results also indicate a lack of inclusion of psychosocial aspects in routine care, which emphasizes opportunities for necessary improvements.

## Data Availability

Data from the study are not publicly available due to data protection rules. Data are available from the corresponding author upon reasonable request and after approval by the local data protection officer.

## Abbreviations

HCP: Health care professional
ZAPA: Fragebogen zur Zufriedenheit in der ambulanten Versorgung, a short instrument to assess satisfaction
ZUF-8: Fragebogen zur Messung der Patientenzufriedenheit, a short instrument to assess patient satisfaction with care
